# Network Brain-Computer Interface (nBCI): An Alternative Approach for Cognitive Prosthetics

**DOI:** 10.3389/fnins.2018.00790

**Published:** 2018-11-01

**Authors:** Vivek P. Buch, Andrew G. Richardson, Cameron Brandon, Jennifer Stiso, Monica N. Khattak, Danielle S. Bassett, Timothy H. Lucas

**Affiliations:** ^1^Department of Neurosurgery, Hospital of the University of Pennsylvania, Philadelphia, PA, United States; ^2^Department of Neuroscience, University of Pennsylvania, Philadelphia, PA, United States; ^3^Department of Bioengineering, University of Pennsylvania, Philadelphia, PA, United States; ^4^Department of Physics and Astronomy, University of Pennsylvania, Philadelphia, PA, United States; ^5^Department of Neurology, University of Pennsylvania, Philadelphia, PA, United States; ^6^Department of Electrical and Systems Engineering, University of Pennsylvania, Philadelphia, PA, United States

**Keywords:** network brain-computer interface, cognitive prosthetic, brain-computer interface (BCI), cognitive performance, connectivity, network science

## Abstract

Brain computer interfaces (BCIs) have been applied to sensorimotor systems for many years. However, BCI technology has broad potential beyond sensorimotor systems. The emerging field of cognitive prosthetics, for example, promises to improve learning and memory for patients with cognitive impairment. Unfortunately, our understanding of the neural mechanisms underlying these cognitive processes remains limited in part due to the extensive individual variability in neural coding and circuit function. As a consequence, the development of methods to ascertain optimal control signals for cognitive decoding and restoration remains an active area of inquiry. To advance the field, robust tools are required to quantify time-varying and task-dependent brain states predictive of cognitive performance. Here, we suggest that network science is a natural language in which to formulate and apply such tools. In support of our argument, we offer a simple demonstration of the feasibility of a network approach to BCI control signals, which we refer to as network BCI (nBCI). Finally, in a single subject example, we show that nBCI can reliably predict online cognitive performance and is superior to certain common spectral approaches currently used in BCIs. Our review of the literature and preliminary findings support the notion that nBCI could provide a powerful approach for future applications in cognitive prosthetics.

## Introduction

### The Success of Sensorimotor BCI Technology

Brain computer interfaces (BCIs) utilize neural input functions to control task-oriented systems (Vidal, 1973). Motor BCIs highlight the translational success of this strategy. Motor or efferent BCIs transform neural signals from motor regions into command signals for external effectors such as robotic arms, or for internal effectors such as paralyzed forearm muscles. The success of translating motor BCIs, now in clinical trials, may be attributed to several contributing factors. First, motor decoding algorithms leverage decades of research characterizing the relationships between neural activity and movement behaviors in animal models (Sanes and Donoghue, 1993; Rizzolatti et al., 1996; Hatsopoulos et al., 1998; Kakei et al., 1999; Lu and Ashe, 2005; Li et al., 2015). Movement-related features may be extracted from a number of input sources, including single units, multi-units, and local field potentials (LFP) (Hochberg et al., 2006; Leuthardt et al., 2006). Input sources originate from brain regions with stable relationships to the desired output functions, including limb movements and muscle activations. Finally, movement-related features from neural signals may be extracted during *passive* observation of homologous movements – such as the movements of a robotic arm – just as they would during movement of an intact arm (Jeannerod, 1995; Decety, 1996; Pfurtscheller and Neuper, 1997; Lacourse et al., 2005; Hanakawa et al., 2008; Miller et al., 2010). Consequently, decoding algorithms may be calibrated from the motor regions of a paralyzed patient even though the patient lacks the ability to execute the movement him/herself.

### The Challenges of Cognitive BCI Technology

BCI technology may be applied to other areas of unmet clinical need, such as restoring learning and memory for the cognitively impaired. Unfortunately, the fundamental physiology that underlies the development of sensorimotor BCIs is unlikely to be directly applicable to higher-order cognition. This is due, in part, to the fact that the processes underlying higher-order cognitive functions such as executive function depend upon the dynamic engagement and control of distributed neural circuits (Luna et al., 2001; Gläscher and Büchel, 2005; FitzGerald et al., 2012). Consequently, there is little agreement regarding the ideal brain target from which to derive neural input signals for a cognitive BCI. Nor is there agreement on the ideal input source itself, whether single- or multi-unit spiking, evoked potentials, or spectral profiles of sensor signals. Also, there is broad uncertainty concerning the stability of input signals as they relate to cognitive processes. These challenges are made even more acute by the substratal fact that animal models do not faithfully represent the complexities of human cognition.

Notably, these challenges have not prevented investigators from exploring putative cognitive prosthetic control signals. The best examples are those that attempt to define the neural signature of memory formation. Investigators have used changes in regional LFP oscillatory activity before (Guderian et al., 2009; Fell et al., 2011; Hanslmayr and Staudigl, 2014; Merkow et al., 2014) and during encoding (Klimesch et al., 1997; Fell et al., 2001; Sederberg et al., 2007), as well as temporally precise single unit hippocampal activity (Rutishauser et al., 2010), to predict subsequent recall. Utilizing multimodal analysis of event-related potentials and event-related desynchronization, a recent study was able to successfully predict depth of cognitive processing in memory, language, and visual imagination task domains (Nicolae et al., 2017). In other cognitive realms such as experiential learning, executive control, and dynamic online cognitive performance, less is known about local neural signatures. This may in part be due to the fact that critical regions involved in these higher-order functions are spatially distributed (Luna et al., 2001; Gläscher and Büchel, 2005; FitzGerald et al., 2012) and require coordinated activity (Friston, 1994; Tononi et al., 1994; Büchel et al., 1999; Salinas and Sejnowski, 2001; Kahana, 2006; Chennu et al., 2014; Voytek et al., 2015). Therefore, measuring local, regional, or global metrics of neural activity alone may not provide the ideal control signal for cognitive BCI technology. Rather, the addition of quantitative measures of coordinated activity – often referred to as functional *connectivity* – could improve the reliability and generalizability of the control signal.

### Network Analysis in Cognitive Function

Network neuroscience is an emerging discipline that enables analysis of distributed, dynamical neural systems (Tononi et al., 1994; Sporns, 2002; Bullmore and Sporns, 2009; Rubinov and Sporns, 2010; Sporns, 2011) through a suite of flexible and generalizable mathematical tools borrowed from mathematics (graph theory), physics (statistical mechanics), computer science, and engineering. Networks are described in terms of neural elements (nodes) and the connections between them (edges). Notably, any edge between node *i* and node *j* can be assigned a continuous value of weight, which captures the strength of the connection between node *i* and node *j*. The pattern of weights across edges connecting nodes is frequently referred to as the network’s topology (Rubinov and Sporns, 2010). Network analysis tools can utilize structural (tractography) or functional (physiology) data sources to construct different sorts of networks. Networks composed of functional connections (or edges) are referred to as functional networks, and networks composed of structural connections (or edges) are referred to as structural networks. Functional networks can be constructed from data acquired either during the resting state or during the performance of a task.

During the performance of increasingly cognitively demanding tasks, oscillatory signals are thought to impart critical information regarding task-relevant neural activity in regions activated by the task (Friston, 1994; Rubinov and Sporns, 2010; Burke et al., 2015a). Functional connectivity (FC) can be measured through frequency-specific synchronization, or phase-locking (Luauté et al., 2015), of two oscillating neural sources (Bamdad et al., 2015; Luauté et al., 2015; Ezzyat et al., 2017; Jiang et al., 2017; Ezzyat et al., 2018). Two regions displaying phase synchrony, a statistical relation between the instantaneous phases of the signals in both areas, are often interpreted as being functionally connected. Ensemble synchrony is thought to be an important mechanism underlying cognitive processing (Salinas and Sejnowski, 2001; deBettencourt et al., 2015; Kucewicz et al., 2018), and the loss of synchrony is implicated in cognitive disease states such as dementia and schizophrenia (Burke et al., 2015b; Jacobs et al., 2016; Merkow et al., 2017; Ortner et al., 2017). More generally, functional connectivity reflects pairwise statistical relationships that can display complex patterns indicative of non-trivial network topologies. The features of the functional network can be quantified using numerous statistics commonly referred to as graph statistics, network measures, or connectomic metrics. The quantification of network architecture in patterns of FC can provide robust estimates of task-dependent interregional coordination, and its relation to multisensory processing, cognition, memory, and learning (Friston, 1997; Tononi, 1998; Sporns et al., 2004; Miller et al., 2007; Canolty et al., 2009; Bassett et al., 2015; McGregor and Gribble, 2017; Nicolae et al., 2017).

Here we propose that network approaches to distilling the topology of functional connectivity patterns could offer an improved framework for creating BCI control signals for the cognitive domain. Below we discuss the approach taken by current cognitive BCI systems and provide preliminary evidence for the utility, feasibility, and potential superiority of a network BCI (nBCI) approach.

## Current Advances

### Advances in Cognitive BCI

To date, experiments with cognitive BCIs have focused on improving memory, attention, and consciousness (Bamdad et al., 2015; Burke et al., 2015a; deBettencourt et al., 2015; Luauté et al., 2015; Ezzyat et al., 2017, 2018; Jiang et al., 2017; Ortner et al., 2017; Kucewicz et al., 2018). Burke et al. (2015a) attempted an initial memory BCI system using theta (4–8 Hz) and alpha (9–14 Hz) band spectral activity to trigger the optimal timing of object presentation during memory encoding. The authors found that features of theta and alpha band spectral activity did not constitute reliable control signals across sessions or across subjects (Burke et al., 2015a). Subsequent improvements in defining the control signal have employed supervised multivariate pattern analysis techniques to assess global spectral activity that correlates with superior and inferior encoding states within each subject (Ezzyat et al., 2017). Initial stimulation paradigms delivered during randomly assigned memory encoding epochs resulted in inconsistent behavioral effects (Jacobs et al., 2016; Ezzyat et al., 2017; Merkow et al., 2017). However, recent studies demonstrate that short bursts of lateral temporal cortex stimulation delivered only during poor encoding states are associated with a disruption of the innate spectral activity and an improvement in memory performance (Ezzyat et al., 2018; Kucewicz et al., 2018). In non-memory cognitive systems, control signals utilizing blood-oxygen-level dependent (BOLD), evoked potentials, or spectral analysis have been incorporated as biofeedback therapy for self-optimization of attentional networks (deBettencourt et al., 2015; Jiang et al., 2017) and as communicative tools for patients with altered levels of consciousness (Luauté et al., 2015; Ortner et al., 2017). These approaches have also been used to assess and predict the depth of cognitive processing, reflected by the level of task difficulty, in memory, language, and visual task domains (Nicolae et al., 2017).

Though current systems provide a foundation for cognitive BCI technology, there is difficulty extending these approaches to other realms of cognitive function such as experiential learning and executive control, or to adaptive contexts in which one wishes to optimize dynamic online cognitive performance in a singularly demanding task. This difficulty is due in part to a lack of spatiotemporally resolved BOLD, evoked, or oscillatory neural signals whose features reliably predict individual differences in these other areas of cognitive function. Furthermore, although a certain profile of spectral power, namely the spectral tilt phenomenon, is thought to reflect cortical activation (Miller et al., 2007; Burke et al., 2015b), analyzing multifocal spectral activity is not a quantitative measure of regional interactions. For dynamic cognitive tasks, precisely quantifying these interactions may be more task relevant than the collective regional pattern of activity (Varela, 1995; Friston, 1997; Tononi, 1998; Büchel et al., 1999; Sporns et al., 2004; Rypma et al., 2006; Canolty et al., 2009; Brancucci, 2012; Bassett et al., 2015; McGregor and Gribble, 2017).

### Network Analysis Applied to Current BCI Systems

The concept of using phase-based FC as a putative control signal for BCI technology has been examined in a limited number of studies focused on motor imagery (Brunner et al., 2006; Carreiras et al., 2012; Billinger et al., 2013), motor execution (Billinger et al., 2015), and the visually evoked P300 speller response(Kabbara et al., 2016). All of these systems utilize non-invasive electroencephalography (EEG) as their neural input signal and phase-locking (Lachaux et al., 1999) as their FC metric; and all report improvement in BCI functionality with the incorporation of FC features. In a simple reach and saccade cognitive task Courellis et al. (2017), extract statistical dependencies of EEG region of interest time series data to demonstrate the plausibility of cognitive network detection using a non-invasive BCI. Using intracranial EEG, phase-based network analysis of high gamma (>70 Hz) synchrony has recently been shown to accurately distinguish wakeful versus sleep states (Mikulan et al., 2018). The potential to also incorporate complex network metrics has been examined in a simple finger-tapping task (Daly et al., 2012). This study assessed the discrimination ability between tap versus no-tap trials using the clustering coefficient, a graph metric of local network integration. The authors report a superior detection capability of tap versus no-tap trials when compared to traditional event-related EEG metrics of synchronization or desynchronization. However, this study was only a feasibility study of the analytical technique and did not actually utilize complex network metrics in a BCI system.

## Future Directions

### Proposing nBCI for the Cognitive Domain

Here we propose the incorporation of network analysis as a potential control signal for cognitive BCI systems. Global measures of network organization can provide insight into brain states during cognitive tasks (Palva et al., 2005; Besserve et al., 2008). One noted example of such a measure is network strength, which can be calculated by averaging the sum of edge weights from each node in a network. Furthermore, metrics quantifying the mesoscale integration or segregation states of functional circuits or networks include the clustering coefficient and the modularity. These two metrics have been shown to correlate with learning and cognitive performance over long time-scales (Husken et al., 2002; Bullmore and Sporns, 2009; Bassett et al., 2011; Hermundstad et al., 2011; Ellefsen et al., 2015). Anatomically, there is evidence for flexible hubs in dorsal and ventral frontoparietal networks and cingulo-opercular networks that have strong influence on saliency and cognitive control (Corbetta and Shulman, 2002; Cole et al., 2010, 2013; Power et al., 2011; Power and Petersen, 2013). Network metrics that account for the paths by which information can be transmitted in the network, such as betweenness centrality and communicability, may also provide meaningful insight regarding these types of regional influences on network information flow and brain state transitions (Borgatti, 2005; Newman, 2005; Estrada and Hatano, 2008; Crofts and Higham, 2009; Betzel et al., 2016). Therefore, these metrics may hold promise as a potential feature vector for predicting high performing brain states in otherwise difficult-to-decode neural data. For network metrics to constitute a plausible control signal, dynamic changes in network metrics must occur on short time-scales and must predict upcoming cognitive performance, allowing for real-time control of a neuromodulatory system.

### Preliminary Results

Using single-trial phase locking statistics (Lachaux et al., 2000) we provide preliminary evidence in a single human subject on the use of dynamic changes in functional brain network statistics as a predictive signal for online cognitive performance. This subject was undergoing clinical monitoring for refractory epilepsy with stereotactically placed intracranial EEG (sEEG). A total of 122 electrode contacts located diffusely within bilateral frontal, temporal, and parietal cortical and subcortical regions, as well as deep limbic structures were used as individual nodes in our network analysis. We used a simple temporal expectancy reaction time (RT) task to assess online cognitive performance. Temporal expectancy engages several distinct cognitive processes such as those involved in saliency (Coull and Nobre, 1998), attention (Tecce, 1971, 1972; Colombo and Richman, 2002), temporal processing (Onoe et al., 2001; Mauk and Buonomano, 2004), and plasticity (Dallérac et al., 2017); with induced changes in regional electrophysiology (Walter et al., 1964; Funderud et al., 2012) and network-wide functional imaging (Onoe et al., 2001; Nagai et al., 2004). Trials consisted of a visual *cue* signal (a white box presented on a black screen), an instructed delay period (500 or 1500 ms randomly chosen with equal probability), and a *go* signal (a color change of box from white to yellow) after which the subject made a keypress to indicate the perception of the *go* signal. The RT was defined as the time between *go* and keypress. For the purposes of this report, only trials with a 1500-ms delay period were analyzed (*n* = 57 trials).

**FIGURE 1:**
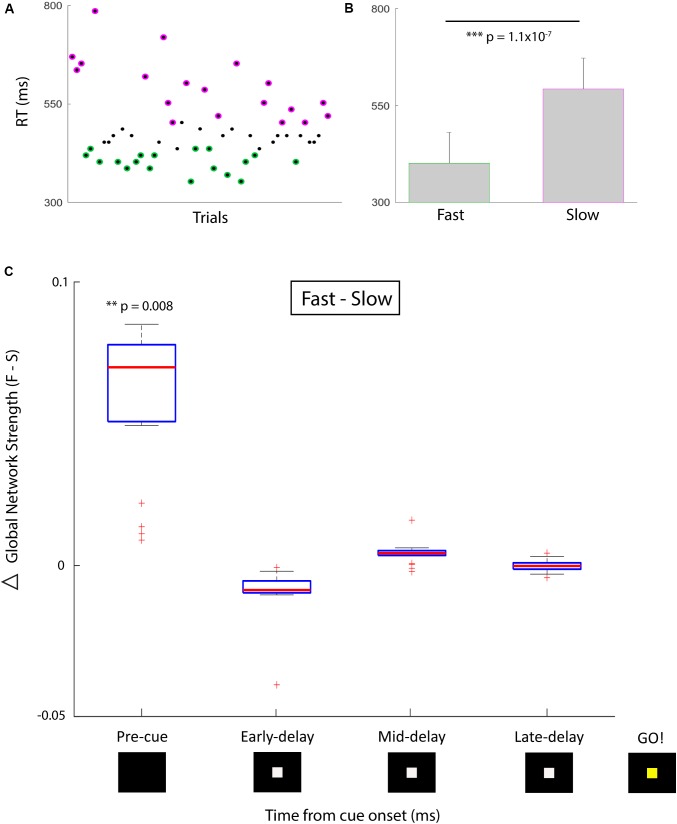
Behavioral performance and correlative network strength over time. **(A)** Simple temporal expectancy task reaction time (RT) per trial. Fastest third (green) and slowest third (magenta) of trials were determined. **(B)** Mean RTs for the fast versus slow trial groups were significantly different. **(C)** Global network strength (PLV) over time for fast (first plot) versus slow (second plot) trials. Box plots indicate median and interquartile range, whiskers indicate 95% data coverage. Timing is centered around cue presentation (0 ms). First time bin signifies *pre-cue* period (-500 to 0 ms), followed by three 500 ms delay period bins. After a 1500 ms delay, the cue changes color which designates the *go* signal. There was a significant difference in the *pre-cue* period for global network strength between the distribution of fast and slow trials. Asterisk denote degree of significance coinciding with p values.

The phase-locking value (PLV) (Lachaux et al., 1999) for high gamma (70–100 Hz) activity between all pairs of electrodes was computed on each trial in sliding 500 ms time bins, using the Hilbert transform to extract instantaneous phase. For the network analysis, each sEEG electrode represented a network node and the edge weight between nodes was defined by the high gamma (70–100 Hz) PLV for that pair. Global network strength, a measure of ensemble synchrony, was computed by taking the average edge weight across all pairwise nodal interactions in the network. We divided trials based on ‘good’ (fast RT, *n* = 19) and ‘poor’ (slow RT, *n* = 19) performance such that the mean RT was significantly different in the two conditions (Figures 1A,B; one-sided Wilcoxon rank sum test, *z* = -5.2, *p* = 1.1 × 10^-7^). We compared global network strength across four 500-ms time bins between the two conditions: *pre-cue* (-500 to 0 ms relative to *cue*), *early delay* (0–500 ms), *mid-delay* (500–1000 ms), and *late-delay* (1000–1500 ms). We found that in the *pre-cue* period, fast-RT trials were characterized by significantly higher global network strength than slow-RT trials (Figure 1C; two-sided Wilcoxon rank sum test, *z* = 2.6, *p* = 0.008, multiple comparison threshold *p* = 0.0125). There was no significant difference in global network strength between performance conditions for the three delay time bins.

**FIGURE 2:**
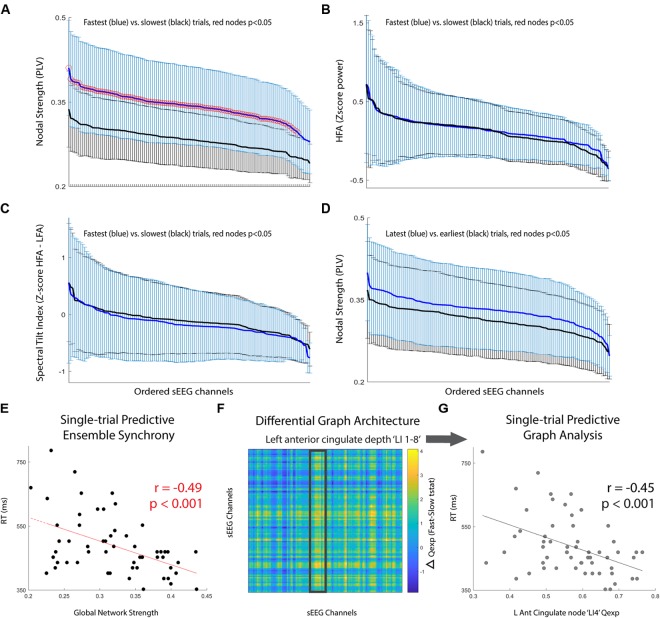
*Pre-cue* period network analysis. (**A–C)** Comparison of mean +/- std for fastest (blue) versus slowest (black) trials. Metrics used are **(A)** nodal strength, **(B)** high frequency (70–100 Hz) spectral power, and **(C)** spectral tilt (HFA-LFA), respectively. Nodal strength, a network statistical measure of average synchrony between that node and all others, shows significantly and globally increased *pre-cue* values in fast compared to slow trials. **(D)** Comparison of mean +/- std for latest (blue) versus earliest (black) trials using nodal strength. No significant difference for any particular node, however, there was a trend toward increasing mean nodal strength for latest compared to earliest trials (*p* = 0.08). **(E)** Global network strength in the *pre-cue* period corresponding to each upcoming trial and associated trial reaction time. There was a significant predictive correlation. **(F)** Difference in network communicability during the *pre-cue* period averaged across all fast-slow RT trials; the most significant increase was seen in the left anterior cingulate lead (highlighted box, max channel ‘LI4’ F-S difference t(35) = 2.50, *p* = 0.02). **(G)** Single-trial RT as a function of left anterior cingulate node ‘LI4’ single-trial communicability demonstrating independent predictive correlation.

Next, we compared the performance discrimination ability of nodal strength (network strength per node) to traditional spectral control signals for the *pre-cue* period: (i) high-frequency activity (HFA; 70–100 Hz power), and (ii) spectral tilt (70–100 Hz z score power minus 3–12 Hz z score power). We found that for nearly all sEEG channels, *pre-cue* nodal strength was significantly higher for fast-RT trials than for slow-RT trials (Figure 2A; *t*(35) = 3.83, *p* = 5.1 × 10^-4^ for the node with maximum difference). This predictive discriminatory ability of subsequent performance was not present for HFA or spectral tilt (Figures 2B,C). Trials were then separated into ‘early’ (first third of trials, *n* = 19) versus ‘late’ (last third of trials, *n* = 19) conditions. There was no significant difference in strength between these conditions at any node (Figure 2D; *t*(41) = 1.71, *p* = 0.09 for the node with maximum difference).

Finally, we computed single-trial *pre-cue* global network strength and compared it to single-trial reaction time. There was a significant correlation between these two variables (Figure 2E; *r* = -0.49, *p* = 1.3 × 10^-4^). We then calculated the communicability (Qexp), a network metric given by the normalized matrix exponential of each single-trial weighted connectivity matrix (Estrada and Hatano, 2008; Crofts and Higham, 2009). Intuitively, communicability is the sum of all possible walks (and thus paths) between two nodes, exponentially down-weighted as length increases (accounting for the decreasing statistical relationship as length increases). The average communicability for a particular node *k* therefore quantifies the relative degree to which each node *k* contributes to walks (and thus paths) of different lengths (Estrada and Hatano, 2008; Crofts and Higham, 2009). We found that communicability was dynamic, and that high *Qexp* in the left anterior cingulate depth electrode during the *pre-cue* period was significantly correlated with the average difference in fast versus slow RT trials (Figure 2F, highlighted region ‘LI 1-8’, maximum nodal F-S difference ‘LI4’ *t*(35) = 2.50, *p* = 0.02). We then found that the single-trial communicability in the left anterior cingulate lead ‘LI4’ independently predicted RT (Figure 2G; *r* = -0.45, *p* = 4.6 × 10^-4^).

To assess the decoding ability of a feature space consisting of *pre-cue* high gamma global network strength and ‘LI4’ communicability, a support vector machine (SVM) was trained and cross-validated for binary classification of ‘Fast’ versus ‘Slow’ RT trials. Significance of 10-fold cross-validated classifier performance was assessed using a permutation test in which binary (‘Fast’ versus ‘Slow’) class labels were randomly generated, area under the curve (AUC) of classifier performance was calculated, and the randomization was repeated 1000 times to create a null distribution of AUCs. The SVM classifier for this single subject achieved reliable subsequent performance prediction (AUC = 0.72, *p* = 0.03).

These are the first data reporting single-trial ensemble synchrony and network metrics as predictive features for online cognitive performance; and they demonstrate superiority over certain common spectral approaches for quantifying behaviorally relevant neural activity. Interestingly, high gamma synchrony has been shown to be correlated with increased wakefulness (Mikulan et al., 2018), providing a possible mechanistic link for the enhanced performance predicted by high gamma ensemble synchrony in our subject. Further, we find dynamic communicability in the left anterior cingulate lead is also predictive of subsequent performance, perhaps consistent with previous evidence of a primary role for cingulo-opercular network influence on cognitive control (Corbetta and Shulman, 2002; Cole et al., 2010; Power and Petersen, 2013).

## Conclusion

Though disorders of cognition are vast, network analyses of functional connectivity may provide meaningful quantifications and correlates of functional impairments as well as ready classifications of aberrant underlying neural processes (Voytek and Knight, 2015; Cohen and D’Esposito, 2016). We find that in this single human subject example of online cognitive performance, the network analysis approach (nBCI) outperforms certain traditional spectral approaches in antecedent, dynamic performance discrimination. Furthermore, using the Hilbert transformation-based PLV to create single-trial weighted networks provides a computationally lightweight methodology for potential real-time decoding and feature extraction. These data preliminarily support our proposed notion that network analysis could be applied as a control signal in cognitive BCI systems. As a rapidly quantifiable analysis of network interactions, nBCI may enable increased generalizability of BCI technology for cognitive rehabilitation.

## Ethics Statement

This study was carried out in accordance with the recommendations of the Human Subjects Research guidelines by the University of Pennsylvania’s Office of Regulatory Affairs, Institutional Review Board. The protocol was approved by the University of Pennsylvania Institutional Review Board. All subjects gave written informed consent in accordance with the Declaration of Helsinki.

## Author Contributions

VB formulated the concept, performed the data analysis, and primarily wrote the paper. AR helped in the organization and writing of the paper and figures. CB was responsible for the data acquisition. JS guided the connectivity analysis. MK helped with the literature review. DB and TL provided their expertise to help frame the proposed idea as well as provided a detailed review of the paper.

## Conflict of Interest Statement

The authors declare that the research was conducted in the absence of any commercial or financial relationships that could be construed as a potential conflict of interest.
